# Development and validation of a method for precise dating of female puberty in laboratory rodents: The puberty ovarian maturation score (Pub-Score)

**DOI:** 10.1038/srep46381

**Published:** 2017-04-12

**Authors:** Francisco Gaytan, Concepción Morales, Silvia Leon, Violeta Heras, Alexia Barroso, Maria S. Avendaño, Maria J. Vazquez, Juan M. Castellano, Juan Roa, Manuel Tena-Sempere

**Affiliations:** 1 Department of Cell Biology, Physiology and Immunology, University of Córdoba, 14004 Cordoba, Spain; 2 Instituto Maimónides de Investigación Biomédica de Córdoba & Hospital Universitario Reina Sofia (IMIBIC/HURS), 14004 Cordoba, Spain; 3 CIBER Fisiopatología de la Obesidad y Nutrición, Instituto de Salud Carlos III, 14004 Cordoba, Spain; 4 Department of Pathology, University of Córdoba, 14004 Córdoba, Spain; 5 FiDiPro Program, Department of Physiology, University of Turku, FIN-20520 Turku, Finland

## Abstract

Puberty is a key developmental event whose primary regulatory mechanisms remain poorly understood. Precise dating of puberty is crucial for experimental (preclinical) studies on its complex neuroendocrine controlling networks. In female laboratory rodents, external signs of puberty, such as vaginal opening (VO) and epithelial cell cornification (i.e., first vaginal estrus, FE), are indirectly related to the maturational state of the ovary and first ovulation, which is the unequivocal marker of puberty. Whereas in rats, VO and FE are almost simultaneous with the first ovulation, these events are not so closely associated in mice. Moreover, external signs of puberty can be uncoupled with first ovulation in both species under certain experimental conditions. We propose herein the Pubertal Ovarian Maturation Score (Pub-score), as novel, reliable method to assess peripubertal ovarian maturation in rats and mice. This method is founded on histological evaluation of pre-pubertal ovarian maturation, based on antral follicle development, and the precise timing of first ovulation, by retrospective dating of maturational and regressive changes in corpora lutea. This approach allows exact timing of puberty within a time-window of at least two weeks after VO in both species, thus facilitating the identification and precise dating of advanced or delayed puberty under various experimental conditions.

Puberty, as the developmental stage when reproductive capacity is achieved and sexual maturation completed, is a crucial event in the lifespan. Timing of puberty, defined by the full (re)-awakening of the elements of the hypothalamic-pituitary-gonadal (HPG) axis, is under the control of numerous internal and external cues, and hence is determined by the complex interplay of genetic and environmental factors^1^,^2^,^3^. In fact, the age of puberty is considered as a sensor (and eventual sentinel) for perturbations of such gene-environment interactions during early developmental periods^4^. Furthermore, epidemiological studies have documented secular changes in the tempo of puberty onset in humans^4^, and suggested a link between alterations in the timing of puberty and a wide range of adverse health outcomes in adult life^5^. In this context, extensive experimentation, using preclinical models, has been conducted in the last decades to elucidate the mechanistic basis of puberty and its eventual alterations^1^. Hence, precise dating of puberty in these models appears essential for proper analysis of the influence of complex neural networks and signals controlling pubertal development, as well as for the detection of alterations (either advancement or delay) in the chronology of such developmental processes, in different experimental conditions.

Several indirect signs of puberty, accessible via non- or minimally-invasive procedures, such as the age of vaginal opening (VO), of the first appearance of cornified epithelial cells in the vagina (i.e., first vaginal estrus; FE), or the presence of a vaginal plug (VP) after mating, have been used as external markers of puberty onset in laboratory rodents^6^. Importantly, both VO and FE are indirect markers of puberty, as they are caused by the rise in estradiol levels during peripubertal period, associated to the first wave of follicular maturation, and can be induced in juvenile rats by estrogen administration^6^. Yet, it is the first ovulation the event that truly represents the end-point of a series of morphological and functional changes at different levels of the HPG axis, and hence constitutes the unequivocal sign that puberty has been achieved.

Rats and mice are the most widely used models in biomedical research. Although they display similar ultra-short estrous cycles, relevant differences exist between these two rodent species regarding the sequence of puberty events. In rats, it has been clearly established that VO and FE are tightly coupled with the first ovulation, and consequently, the age of VO is an indirect, but reliable, marker of puberty onset under physiological conditions^6^. However, these events might be uncoupled under certain experimental conditions, as reported after nutritional^7^ or pharmacological^8^ manipulations. Alike, subtle differences in environmental conditions may also cause some dissociation between these parameters^9^. Therefore, coupling of VO or vaginal epithelial cell cornification with the first ovulation might not be directly assumed under certain experimental settings in rats.

The scenario is far more complicated in mice, in which VO and FE are not so clearly coupled to the first ovulation^10^. Thus, whereas the presence of cornified epithelial cells in adult cycling mice is normally associated with ovulation, this is not necessarily the case in pre-pubertal mice. Thus, in a previous study, Safranski and co-workers aimed to analyze the relationship between VO, FE and post-mating vaginal plug (VP) in mice; they reported that none of the animals had ovulated at the age of VO, only 15% did at the age of FE, whereas 91% had ovulated at the age of VP^10^. Though the presence of VP appears as the most accurate external sign of ovulation, it requires mating at first estrus, which is obviously undesirable in most experimental designs. Even the recommended use of vasectomized stud males is also inadequate, due to the subsequent induction of pseudo-pregnancy. Overall, the data available indicate that while external signs, such as VO or FE, are useful to monitor the rise of estrogens associated to pubertal maturation, they may not be fully reliable for the precise timing of first ovulation in mice, even under physiological conditions.

In this context, it is clear that ovarian histological examination for the occurrence of the first ovulation constitutes the only definitive evidence of puberty completion in both species. This approach provides additional information about the number and structural features of corpora lutea and the proper ovulatory process. However, simple ovarian histology provides a limited, binary (i.e., whether ovulation has occurred or not) information about pubertal ovarian maturation. We propose herein a novel method (that we name *Pub-score*) to assess pubertal development based on the antral follicle developmental stages for still non-ovulating animals, and retrospective timing of the first ovulation in already ovulating ones, based on the dating of corpora lutea (CL) through evaluation of the maturational and regressive steps of functional and regressing CL. This method also provides a wide time window to detect alterations (either delay or advancement) of pubertal timing in animals submitted to pharmacological or genetic manipulations.

## Results

The Pub-score method for pubertal timing is based on antral follicle development in pre-pubertal (still non-ovulating) animals, and on the dating of corpus luteum (CL) maturation and aging in already ovulating ones, thus allowing retrospective timing of puberty. As subtle, but significant differences exist between rats and mice, we will consider these species independently.

### Pub-score in rats

**Figure 1:**
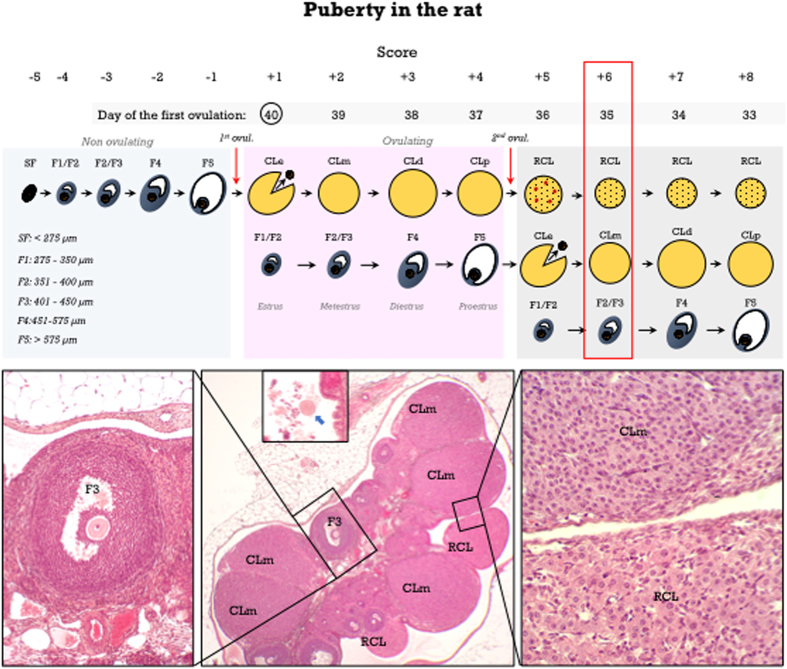
Peripubertal ovarian maturation in the rat. A schematic drawing is presented showing antral follicle development in non-ovulating rats and the first two cycles after the first ovulation, as well as the corresponding Pub-scores from −5 to +8. The age of the animals is matched to the day of the first ovulation (PND-40 in the example; encircled). An ovarian section (stained with hematoxylin and eosin) showing two CL generations, those of the current cycle (two days of age; metestrus; CLm) and regressing CL (CLR), together with eggs at the isthmus (blue arrow in the inset) and class F3 follicles. In accordance, a Pub-score of +6 is assigned and the first ovulation occurred at PND-35 (*red box*).

A schematic drawing of ovarian maturation in peri-pubertal rats is shown in Fig. 1. The first ovulation, occurring at the early first estrus was considered as age-point 0, determining the status of either non-ovulating (negative scores) or ovulating (positive scores) rats.

#### Pub-score for non-ovulating rats

**Figure 2:**
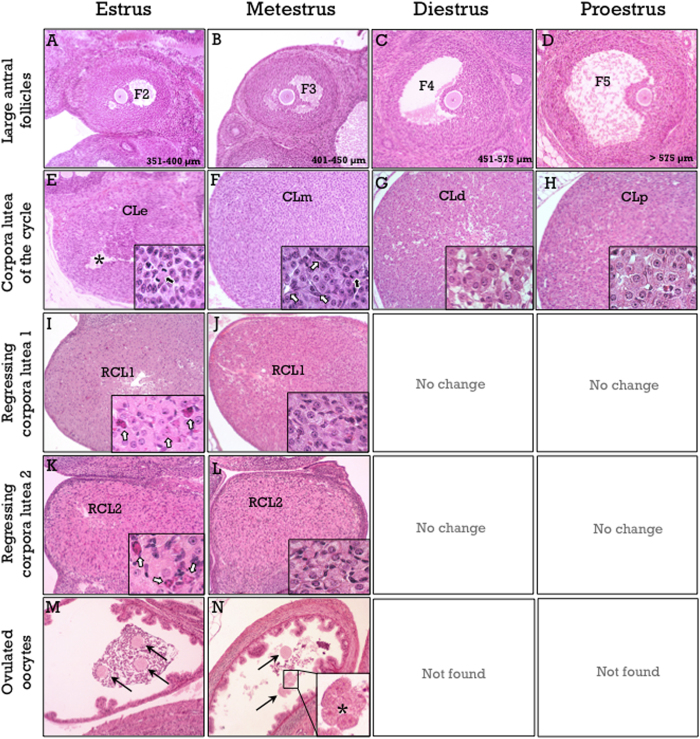
Hallmarks of the rat estrous cycle. These include large antral follicles (**A**–**D**), corpora lutea of the current cycle (**E**–**H**), one cycle-old (**I**–**J**) or two cycle-old (**K**–**L**) regressing corpora lutea, and presence of ovulated oocytes in the oviduct (**M**–**N**). Corpora lutea of the current cycle show non-luteinized granulosa cells and mitotic figures in estrus (arrow in the **E**, *inset*), non- fully luteinized cells in metestrus (**F**), with a prominent vascular pattern and mitotic figures (white and black arrows respectively in the *inset*), and full luteinization in diestrus (**G**) and proestrus (**H**). The presence of class F5 follicles is a discriminating feature between diestrus and proestrus. The first generation of regressing corpora lutea (RCL1) show abundant apoptotic cells in estrus (arrows in the *inset* in panel I), and increased ratio of stromal to steroidogenic cells from metestrus (**J**) to proestrus. The second generations of regressing corpora lutea (RCL2) show a new round of apoptotic cells in estrus (arrows in the *inset* in panel K) and further increased ratio of stromal to steroidogenic cells from metestrus to proestrus. A specific signal for estrus is the presence of cumulus-oocyte-complexes (arrows in **M**) at the ampulla of the oviduct, whereas nearly nude and even fragmented (asterisk in the *inset* in **N**) oocytes in the oviductal isthmus. Oocytes are usually not found in the oviduct thereafter. Hematoxylin and eosin staining.

The most advanced healthy antral follicle class, from small follicles measuring less than 275 μm in diameter to antral follicle classes F1 to F5 (Fig. 2A–D) was determined, and Pub-scores were assigned from −5 to −1 (see legend of Fig. 1 for details). These follicle classes correspond to the final antral follicle growth during the estrous cycle^11^,^12^, and have been used in previous studies to assess pubertal development^13^.

#### Pub-score for ovulating rats

In ovulating rats, dating of the CL of the current cycle in one day-based intervals provides an accurate estimation of the time elapsed from ovulation. *One-day-old CL* (i.e., in estrus) show the rupture site at the ovarian surface and still non-luteinized granulosa cells (Fig. 2E). One additional unequivocal signal for this stage is the presence of cumulus-oocyte complexes in the dilated ampulla of the oviduct (Fig. 2M). This would correspond to a Pub-score of +1. *Two day-old CL* (i.e., in metestrus) display non-fully luteinized granulosa cells, increasing vascularization with a marked vascular pattern, and abundant mitotic figures (Fig. 2F). Additional signs are the presence of nude (even fragmented) oocytes in the oviductal isthmus (Fig. 2N), as well as class F2/F3 follicles. This stage would correspond to a Pub-score of +2. *Three day-old CL* (i.e., in diestrus) show fully luteinized cells with large cytoplasm (Fig. 2G) and full vascularization, while mitotic figures are nearly absent. An additional feature is the presence of F4 follicles (Fig. 2C), whereas oocytes are usually not found in the oviduct; this would correspond to a Pub-score of +3. Finally, *four day-old CL* (i.e., in proestrus) have similar morphological features to those of three day-old CL (Fig. 2H), but with the discriminating feature of the presence of preovulatory class F5 follicles (Fig. 2D); this stage would correspond to a Pub-score of +4.

**Figure 3:**
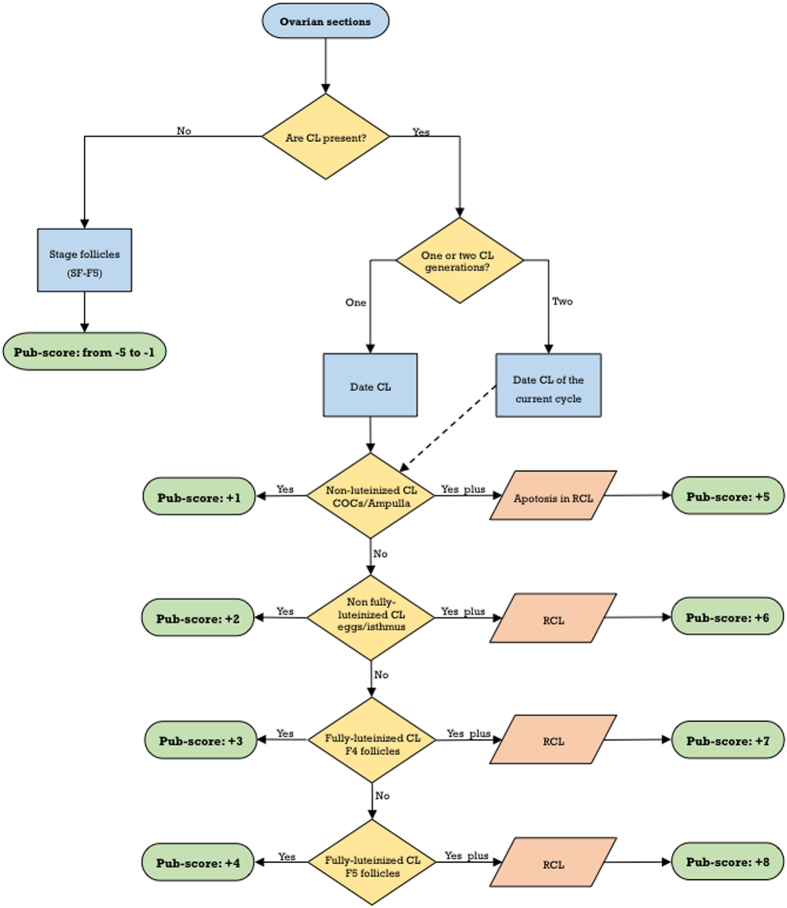
Flowchart for Pub-score assignment in the rat. Criteria for Pub-scores from −5 to +8 are indicated. Reiteration of this process if two generations of regressing corpora lutea are present, provides Pub-scores from +9 to +12.

The presence of more than one CL generation indicates that rats are in the second estrous cycle. Regressing CL can be easily distinguished from those of the current cycle by the higher proportion of stromal cells, as steroidogenic cells are preferentially eliminated by apoptosis at the proestrus-estrus transition. On the second estrus, i.e., five days after the first ovulation, in addition to the presence of newly formed CL, the CL of the previous cycle show abundant apoptotic cells (Fig. 2I). These regressing CL did not show significant structural changes in the following three days (from metestrus to proestrus), but they undergo a new apoptotic burst at the next proestrus-estrus transition (Fig. 2K). Dating of the CL of the current cycle as already indicated, together with the presence of a generation of regressing CL, determine Pub-scores from +5 to +8. When three CL generations, i.e., those of the current cycle and two generations of regressing CL (Fig. 2I–L) -characterized by an increasing ratio of stromal to steroidogenic cells-, can be identified, Pub-scores from +9 to +12 are assigned. A flowchart for Pub-score assignment in the peripubertal rat ovary is proposed in Fig. 3. Finally, the age at the first ovulation can be estimated from Pub-score data, according to the formula: *Age at first ovulation *=* Age* − *Pub-score* +*1*.

### Pub-score in mice

**Figure 4:**
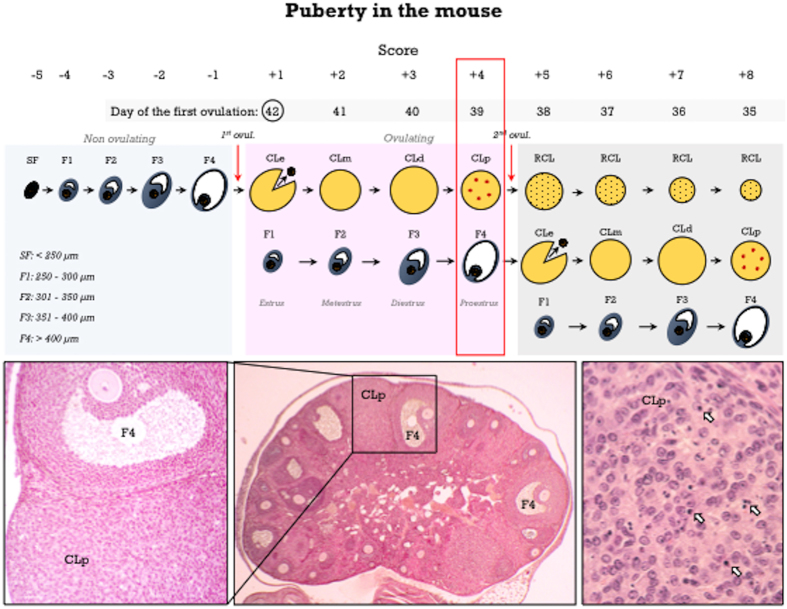
Peripubertal ovarian maturation in the mouse. Schematic drawing showing antral follicle development in non-ovulating mice and the first two cycles after the first ovulation, and the corresponding Pub-scores from −5 to +8. The age of the animals is matched to the day of the first ovulation (PND-42 in the example; encircled). An ovarian section (stained with hematoxylin and eosin) showing corpora lutea of the current cycle (four day-old; CLp) with abundant apoptotic cells (arrows) and class F4 follicles. In accordance, a Pub-score of +4 is assigned and the first ovulation occurred at PND-39 (*red box*).

A schematic drawing of the pubertal maturational changes in the mouse ovary is depicted in Fig. 4. Basic procedure for Pub-score assignment is roughly similar to that for rats, but taking into account some significant differences between both species.

#### Pub-score for non-ovulating mice

**Figure 5:**
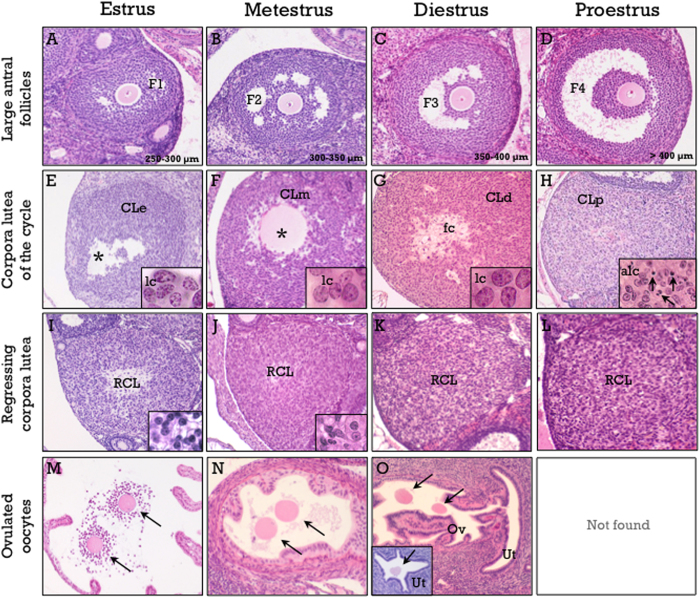
Hallmarks of the mouse estrous cycle. These include large antral follicles (**A**–**D**), corpora lutea of the current cycle (**E**–**H**), one cycle-old (**I**–**L**) regressing corpora lutea, and presence of ovulated oocytes in the oviduct (**M**–**O**). Corpora lutea of the current cycle show non-luteinized granulosa cells (lc) in estrus (CLe in panel E), non-fully luteinized cells in metestrus (CLm in panel F), with a prominent vascular pattern and mitotic figures, and full luteinization in diestrus (CLd in panel G). An empty cavity (denoted by asterisks) is frequently observed in estrus and metestrus, whereas a fibrous center (fc in **G**) is frequent in diestrus. The presence of apoptotic cells is characteristic of the CL in proestrus (arrows in panel H). Regressing corpora lutea (RCL in panels I–L) show an increasing ratio of stromal to steroidogenic cells, progressively decreasing size and are practically demised at the end of the cycle. A specific signal for estrus is the presence of cumulus-oocyte-complexes (arrows in panel M) at the ampulla of the oviduct. Nude oocytes are located at the isthmus in metestrus (arrows in panel N), at the utero-tubal junction (arrows in panel O) or at the tip of the uterine horn (arrow in the inset in **O**) in diestrus, and are not found in proestrus. Hematoxylin and eosin staining.

For scoring purposes, antral follicles were divided into a baseline of small follicles (SF, measuring less than 250 μm) and four discrete follicle classes, from F1 to F4 (Figs 4 and 5A–D). This was achieved by measuring the six largest healthy antral follicles in each day of the estrous cycle, assuming that follicles committed to the next ovulation are included in this cohort (Table 1). These follicle classes have been previously used to assess pubertal development in mice^14^. According to the stage of antral follicle development (from SF to F4), Pub-scores from −5 to −1 are assigned.

#### Pub-score for ovulating mice

**Figure 6:**
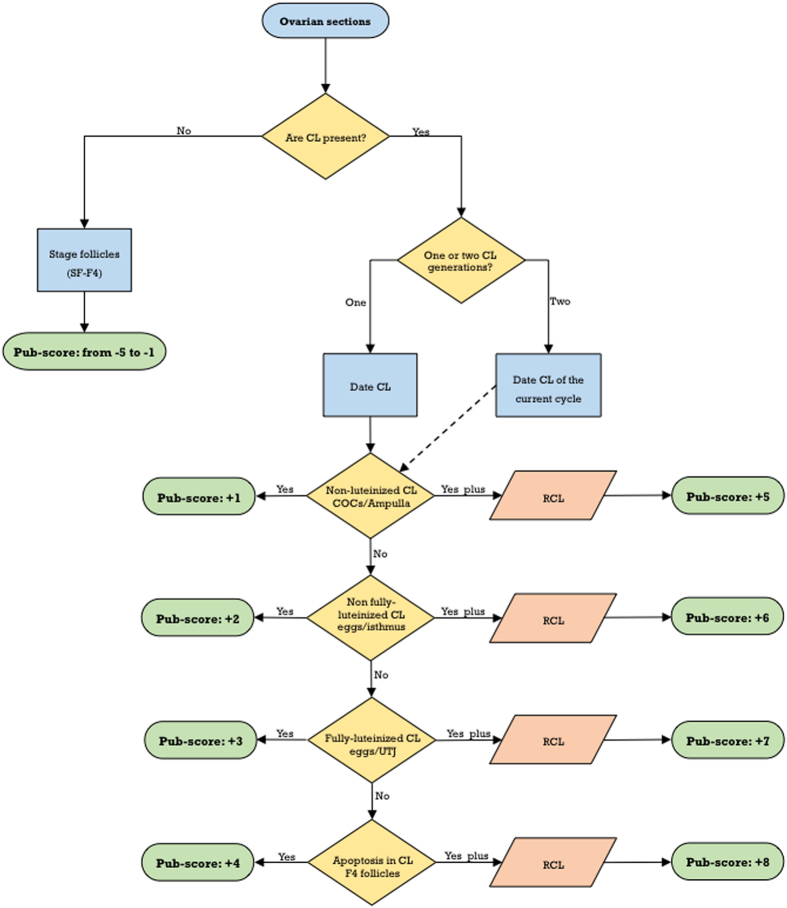
Flowchart for Pub-score assignment in the mouse. Criteria for Pub-scores from −5 to +8 are indicated.

Maturational stages of the CL were quite similar to those in the rat (see Figs 4 and 5 for details). The first day after ovulation (i.e., Pub-score +1) is characterized by newly-formed CL, showing non-luteinized cells (Fig. 5E), and the presence of cumulus-oocyte complexes at the ampulla (Fig. 5M). *Two day-old CL* (Pub-score +2) show non-fully luteinized cells (Fig. 5F), and abundant mitotic figures. Additional features are the presence of F2 follicles, and nude oocytes located at the isthmus (Fig. 5N). *Three day-old CL* (corresponding to diestrus) display full luteinization and a fibrous center (Fig. 5G). Additional signs are the presence of F3 follicles and nude oocytes located at the utero-tubal junction or even in the uterine cavity (Fig. 5O); this stage would correspond to a Pub-score of +3. The landmark for *four day-old CL* (i.e., at proestrus, corresponding to Pub-score +4) is the presence of abundant apoptotic cells (Fig. 5H). This is in contrast with rat CL, which do not show apoptotic cells up to late proestrus-early estrus. An additional feature is the presence of preovulatory F4 follicles. In mouse ovaries showing more than one CL generation, CL of the current cycle should be dated and Pub-scores from +5 to +8 assigned. However, in sharp contrast with the rat, regressing CL are almost completely eliminated in their second estrous cycle (Fig. 5I–L) and, therefore, CL cannot be clearly recognizable beyond 8 days after first ovulation; hence, in mice, Pub-scores higher than +8 cannot be accurately assigned. A flowchart for Pub-score assignment in the mouse is proposed in Fig. 6.

### Applying Pub-score for actual estimation of pubertal timing in rodents

To analyze the relationship between vaginal opening (VO), a widely used external sign of puberty, and the age of the first ovulation (as genuine sign of pubertal occurrence), we performed correlation analyses between the recorded age at VO and the age at first ovulation, estimated using the Pub-score data. Notably, relevant differences were detected between rats and mice with respect to the timing of different puberty markers (Table 2). In rats, VO and first ovulation were almost simultaneous, with a time elapsed of less than one day between both events, which were strongly correlated. In sharp contrast, the first ovulation in mice occurs about 7 days after VO in average. Although a moderate positive correlation between VO and the first ovulation also exist in mice, the age at the first ovulation did show a higher degree of variability.

## Discussion

External signs of puberty onset, such as VO or FE, are widely used to assess pubertal development. However, these indirect signs of puberty might be uncoupled with the first ovulation, in both physiological (specially, in mice) and experimental (in both rats and mice) conditions. Ultimate information about the pubertal maturation status can be only achieved by histological ovarian examination (i.e., by assessing the presence of CL, as morphological evidence that first ovulation has occurred) or by flushing of the oviduct (i.e., by searching for ovulated oocytes). While the presence of oocytes in the oviduct constitutes an unequivocal sign of ovulation, its detection is restricted to the first two days after ovulation, the period when released oocytes remain in the oviduct. On the other hand, usual ovarian examination for the presence of CL provides conclusive evidence for ovulation, but it is limited only to binary information (i.e., whether ovulation has occurred or not), without much additional clues about critical aspects of pubertal maturation, such as its timing.

The Pub-score method that we present here allows the semi-quantitative evaluation of pre-ovulatory ovarian maturation and the precise timing of puberty (i.e., by defining the age at the first ovulation) by retrospective evaluation of CL development and aging, thus determining a relatively wide time-window for comparing puberty age in different conditions in laboratory rodents, including perturbations of internal (e.g., endocrine and metabolic) or external (e.g., social or environmental) cues. Indeed, while for validation purposes, only neutral (control) conditions are presented here, preliminary application of Pub-score in the context of various on-going studies in our group is allowing us to precisely date the timing of puberty in different models of pubertal alteration, including early-onset obesity and subnutrition (*our unpublished data*). It is predictable that other environmental or endogenous modifiers affecting the timing of puberty would equally reflect in altered Pub-scores.

Presentation of the Pub-score data as scatterplots (*see* Suppl. Fig. S1) provides information on the developmental stage of the ovary, the age at the first ovulation, the proportion of ovulating vs. non-ovulating animals, and the dispersion of the data. Considering that the purpose of the present work was to rigorously test and validate the Pub-score method, the ovaries of the animals included in the study were sectioned in whole and all serial sections were analyzed. However, our *a posteriori* analyses confirmed that accurate Pub-score dating of puberty can be achieved by the analysis of just a subset of sections (n = 5) per ovary. This is due to the fact that antral follicles and/or corpora lutea constitute the bulk of the ovary in peripubertal rodents, and hence are present in virtually any ovarian section, thus allowing qualitative staging according to our method. In any event, scoring of the whole ovary would provide valuable additional information, beyond the mere dating of first ovulation, such as quantitative aspects of follicular development and ovulatory dynamics.

According to our data, two relevant differences exist between rats and mice with respect to the correlation between external signs of puberty onset and the age of the first ovulation. First, a close relationship between VO and first ovulation occurs in rats (under physiological conditions), in which both events are synchronized, so that VO can be considered a reliable marker of puberty onset in control rats. In contrast, a variable time gap of about 7 days exists in mice between VO and first ovulation, in agreement with previous reports^10^. The different timing of both events and the higher variability in the age of the first ovulation raises questions about the reliability of vaginal opening as a precise sign of puberty in mice, even under physiological conditions. Second, due to the differences between rats and mice in the process of structural luteolysis (namely, the regressing CL in the rats remain recognizable for at least three cycles while they last only one cycle in mice), the time window for an accurate retrospective timing of the first ovulation is narrower in mice, because accurate identification of CL remnants beyond two cycles is not always possible. In this context, a general rule for both species is that retrospective dating is possible up to two weeks after VO (i.e., dating three cycle-old CL in rats, and two cycle-old CL in mice, as ovulation occurs about 7 days after VO in average (Suppl. Fig. S2).

Of note, the Pub-score method is particularly accurate in rats. The closer relationship between external signs of puberty onset and the first ovulation (at least under physiological conditions), and the protracted CL regression allowing a wider time window for retrospective dating, which add to other advantages, such as the larger body size, favor the use of the rat over mouse models, particularly for endocrine and pubertal/reproductive studies. As additional advantage, the robustness of estrous cycle is clearly higher in rats, whereas in mice it is not unusual to find uneven cycles of 4–5 day length, mainly due to extension of the diestrous phase. In such case, if the early signs of structural luteolysis (normally present at proestrus) are delayed, the score might not exactly fit with the age of the corpus luteum, but would still represent the minimum elapsed time since ovulation. In any event, this minimal mismatch would not substantially affect the pubertal score in mice. Admittedly, however, these potential weaknesses for the use of mice are largely counterbalanced by the limited availability of genetically modified rat models; functional genomics being currently dominated by mouse models. Yet, new efforts and techniques, such as CRISPR-Cas9, are currently in place^15^,^16^, which forecast the routine generation of novel genetically modified rat models that will undoubtedly have a major impact in reproductive research.

A factor to be considered is that, while it allows dating of follicular maturation, the Pub-score cannot conclusively predict the time of the first ovulation in immature rodents that have not reached the ovulatory state, which are scored negatively in our method. This is due to the fact that the first ovulation is not only dependent on the adequate ovarian maturation status, but also on the ability of the hypothalamus-pituitary axis to generate effective pre-ovulatory LH surges. In any event, even in preovulatory rodents, the Pub-score can provide a reliable estimation of the process of follicular development, and hence, of the maturational steps leading to puberty. We admit also that our score method is based on the assumption that immediate prepubertal antral follicle growth is equivalent to antral follicle growth during the estrous cycle. While differences in follicle growth between prepubertal and adult animals have been reported^17^,^18^, these differences seem to affect mainly to early follicle growth during infantile and juvenile periods. Anyway, complete follicle growth reaching preovulatory size (F4 and F5 follicle classes in mice and rats, respectively) is mandatory to maintain a sufficient secretion of estradiol and to undergo positive feedback induction of preovulatory surges and LH-driven responses leading to ovulation (either first or successive ovulations during the lifespan). Thus, as our Pub-score dating method of non-ovulating animals (from −5 to −1) is based on the presence of the largest healthy antral follicles, it is tightly related to the state of ovarian development, therefore providing a semi-quantitative measure of ovarian maturation.

As complex, multi-factorial developmental event, puberty is hallmarked by a series of maturational phenomena, taking place at different levels of the hypothalamic-pituitary-gonadal axis, which define a continuum in the transition from immaturity to reproductive competence^4^. In the case in humans, pubertal maturation is scored on the basis of a combination of different indices, such as those assessing the initiation of breast growth (thelarche), the appearance of pubic hair (pubarche) and gonadal development, including the first menses in girls (menarche). Assessment of the chronology of these indices has permitted identification of intriguing (and worrying) trends for changes in the age of puberty, especially in girls, with a trend for earlier puberty onset (evidenced by earlier thelarche) but no clear anticipation of the age of menarche, which is the external sign of the first ovulation^19^,^20^. Likewise, the non-invasive indices of puberty in rodents, as VO and FE, do provide valuable information on some aspects of the pubertal transition, such as the rise of ovarian estrogens that signals the onset of puberty, as it accompanies pre-pubertal follicular development leading to the first ovulation. However, depending on the rodent species and the experimental conditions, these signs might not be strictly associated to the ovulatory event itself. Moreover, these signs do not permit the close monitoring of timing of pre-pubertal follicular maturation or the follow-up of follicular dynamics once the first ovulation has occurred. These caveats can be covered by complementary approaches allowing a more detailed dating of the different facets of pubertal maturation in rodents. The Pub-score that we propose here is a straightforward method for retrospective dating of female puberty, based in systematic histological examination of the ovary over a time window of approximately two weeks after VO in both rats and mice. This method will allow a more comprehensive and incisive approach in studies on the timing of puberty, in normal and pathophysiological conditions, in conventional preclinical models, using laboratory wild-type and genetically modified rodents.

## Methods

### Animals and tissue processing

Female Wistar rats and CB57BL/6 J mice bred in the vivarium of the University of Cordoba were used. The animals were housed under constant conditions of light (12-h light/dark cycles) and temperature (22 ± 2 °C). The day the litters were born was considered day-1 of age (postnatal day 1; PND-1). Animals were weaned at PND-21, when they were caged in groups of 2–3 animals (all females) and provided with a free access to tap water and standard soy-free chow *ad libitum*. To set the most neutral conditions possible, rats and mice were housed in separate animal rooms, in keeping with standard procedures in our animal facilities. All the experimental protocols were approved by the Córdoba University Ethical Committee of animal experimentation and conducted in accordance with the European Union guidelines for use of experimental animals. Filed data for previously studied Wistar rats were used for classification of estrous-cycle related follicle development and dating of the corpus luteum^11^. For this study, twenty pre-pubertal rats were used for monitoring of vaginal opening and first ovulation. Animals were checked daily by an experienced researcher for vaginal opening from postnatal day 25 (PND-25) onwards. The age at VO was recorded and animals were killed at PND37. In addition, a total of 17 CB57BL/6 J female mice were also checked for VO daily from PND-25 onwards. Depending on the age of VO, the animals were killed between PND-40 and 44. All animals were euthanized in the morning (between 10:00 and 12:00).

After sampling, the ovaries, together with the oviduct and the tip of the uterine horn, were fixed for at least 24 hours in Bouin and processed for paraffin embedding. The ovaries were serially sectioned (10 μm-thick) and stained with hematoxylin and eosin, and viewed under an Elipse 400 microscope.

### Adult cycling mice

Adult (3 month-old), cycling female mice (4–5 per stage of the estrous cycle) were used to assess antral follicle growth and maturational/regressive changes in the CL. For final antral follicle growth determination, the six larger antral follicles found in three animals per cycle stage were measured (with the aid of a micrometer eyepiece incorporated to the microscope), assuming that follicles committed to the next ovulation should be included in this cohort. From these data, we established four discrete follicle classes representing preovulatory follicle growth steps.

### Ovarian observation for Pub-score assignment

For Pub-score assignment, serial ovarian sections were viewed and different variables were considered. Antral follicle classes, the maturation of CL of the current cycle, changes in regressing CL (if present), as well as the location of the oocytes in the oviduct were recorded. Although in this study, for the purpose of rigorous testing and validation of the Pub-score method, the ovaries were sectioned in whole and all serial sections were analyzed, we confirmed by *a posteriori* analyses that evaluation of just a small subset of ovarian sections would suffice for accurate Pub-score dating of puberty. In detail, derived from our validation analysis, it is proposed that the analysis of 5 sections, taken 50 μm (mice) or 100 μm (rats) apart, is sufficient for accurate Pub-score assignment, since antral follicles (in non-ovulating rodents) and corpora lutea (in ovulating animals) constitute the bulk of the ovary at the peripubertal age, and are present in virtually all ovarian sections.

### Statistical analysis

Data are presented as the mean ± SD and the 95% CI intervals were determined (by using Student t distribution values as numbers were below 30). Pearson correlation coefficient was determined to analyze correlation between the age at VO and age at the first ovulation.

## Additional Information

**How to cite this article:** Gaytan, F. *et al*. Development and validation of a method for precise dating of female puberty in laboratory-rodents: The puberty ovarian maturation score (Pub-Score). *Sci. Rep.* **7**, 46381; doi: 10.1038/srep46381 (2017).

**Publisher's note:** Springer Nature remains neutral with regard to jurisdictional claims in published maps and institutional affiliations.

## Supplementary Material

Supplementary Figures

## Figures and Tables

**Table 1:** Mean diameter of the largest healthy antral follicles during the estrous cycle in the mouse.

Stage	Largest antral follicle diameter (μm)		Follicle Class
Estrus	272.2 ± 32.9	95% CI (255.9–288.4)	F1 (250–300 μm)
Metestrus	323.9 ± 28.3	95% CI (309.9–338.0)	F2 (301–350 μm)
Diestrus	372.5 ± 24.1	95% CI (360.0–384.4)	F3 (351–400 μm)
Proestrus	440.5 ± 24.7	95% CI (428.2–452.7)	F4 (>400 μm)

Data correspond to the mean ± SD for N = 18 follicles (measuring the six largest healthy follicles in 3 mice per stage of the cycle). Confidence intervals (CI) are presented in brackets.

**Table 2:** Age (PND) at vaginal opening (VO) and first ovulation (FO), and time elapsed (days) and coefficient of determination (R^2^) between these two parameters, in rats and mice.

Species	N	VO (95% CI)	FO (95% CI)	Time Elapsed (95% CI)	R2
Rat	20	33.2 ± 0.9 (32.7–33.6)	34.1 ± 0.9 (33.7–34.0)	0.8 ± 0.7 (0.5–1.1)	0.54 (*P* < *0.01*)
Mouse	17	31.8 ± 1.8 (30.9–32.7)	38.6 ± 2.8 (37.2–40.0)	6.8 ± 2.3 (5.6–8.0)	0.32 (p < 0.01)

Data are presented as mean ± SD. Confidence intervals (CI) are presented in brackets.
