# Supplementary material for: Development and validation of a method for precise dating of female puberty in laboratory rodents: The puberty ovarian maturation score (Pub-Score)
Source: Sci Rep. 2017 Apr 12;7:46381. doi: 10.1038/srep46381 (PMC5388887; doi:10.1038/srep46381)

Development and validation of a method for precise dating of female puberty in laboratory rodents: The puberty ovarian maturation score (Pub-Score)

Francisco Gaytan[1,2,3], Concepción Morales[4], Silvia Leon[1,2,3], Violeta Heras[1,2,3], Alexia Barroso[1,2,3], Maria S. Avendaño[1,2,3], Maria J. Vazquez[1,2,3], Juan M. Castellano[1,2,3], Juan Roa[1,2,3] and Manuel Tena-Sempere[1,2,3,5]

[1]Department of Cell Biology, Physiology and Immunology, University of Córdoba; [2]Instituto Maimónides de Investigación Biomédica de Córdoba & Hospital Universitario Reina Sofia (IMIBIC/ HURS); [3]CIBER Fisiopatología de la Obesidad y Nutrición, Instituto de Salud Carlos III; and [4]Department of Pathology, University of Córdoba, 14004 Córdoba, Spain; and [5]FiDiPro Program, Department of Physiology, University of Turku, FIN-20520 Turku, Finland

Short Title: Dating puberty in rodents with Pub-Score

Key Words: Puberty, rat, mouse, ovary, cycle, follicle, corpus luteum, dating method

Corresponding authors: Francisco Gaytan (bc1galuf@uco.es)
Manuel Tena-Sempere (fi1tesem@uco.es)
Department of Cell Biology, Physiology & Immunology
Faculty of Medicine, University of Córdoba
Avda. Menéndez Pidal s/n. 14004 Córdoba, SPAIN
FAX: +34-957-218288

Grant Support: This work was supported by grants BFU2011-025021 and BFU2014-57581-P (Ministerio de Economía y Competitividad, Spain; co-funded with EU funds from FEDER Program); PIE-14/00005 (Flexi-Met; Instituto de Salud Carlos III, Ministerio de Sanidad, Spain) Project P12-FQM-01943 (Junta de Andalucía, Spain); and EU research contract REPROBESITY. CIBER Fisiopatología de la Obesidad y Nutrición is an initiative of Instituto de Salud Carlos III.

Disclosure Statement: The authors have nothing to disclose pertaining the contents of this work.

Author Contributions: F.G. and C.M. conducted the histological examinations and primarily defined the scoring method; S.L., V.H., A.B., M.S.A., M.J.V. & J.M.C. participated in the collection of samples and pubertal indices within the course of different experimental studies and discussed the data; J.R. participated in sample collection, discussed the data and prepared the figures; M.T.-S. evaluated and discussed all the data. F.G. & M.T.-S. defined the final scoring method and jointly wrote the paper. All authors revised the manuscript.

Supplemental Figures

Supplemental Figure S1: *Scatterplot presentation of Pub-score data*

An illustrative example of Pub-score dating of female puberty is presented. A group of 10 mice, sampled for histological analysis of the ovaries at PND-40, were subjected to dating following the Pub-score method.

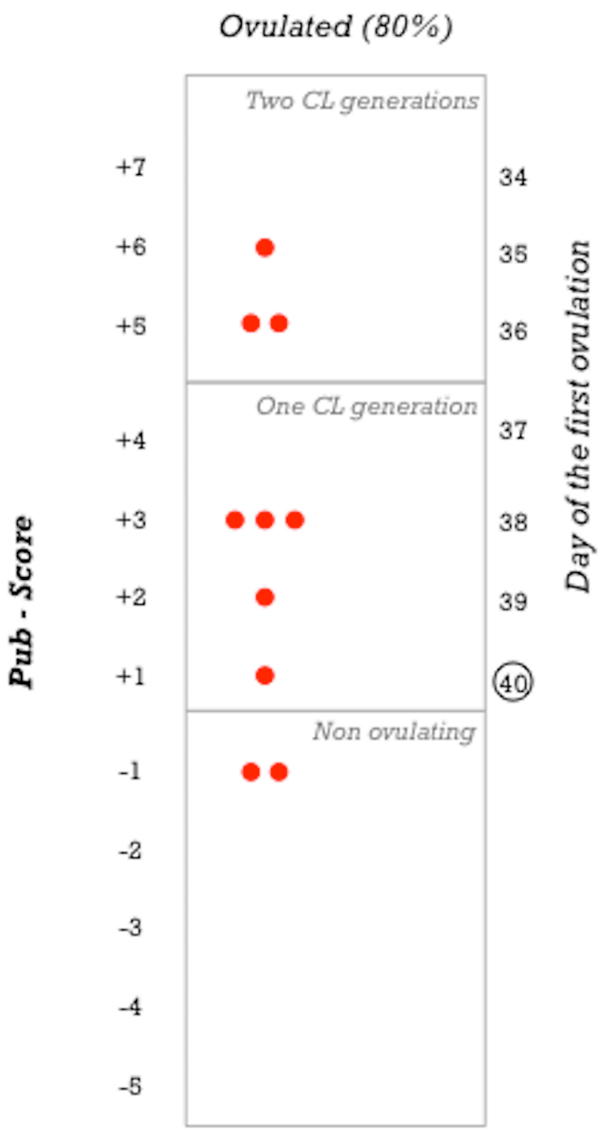

Supplemental Figure S2: *Time window for Pub-score dating of puberty in rodents*
Scheme depicting the time-window for accurate retrospective timing of female puberty, hallmarked by the occurrence of first ovulation, since the age of VO (as external sign of puberty) in rats and mice (*upper* and *lower panels*, respectively).

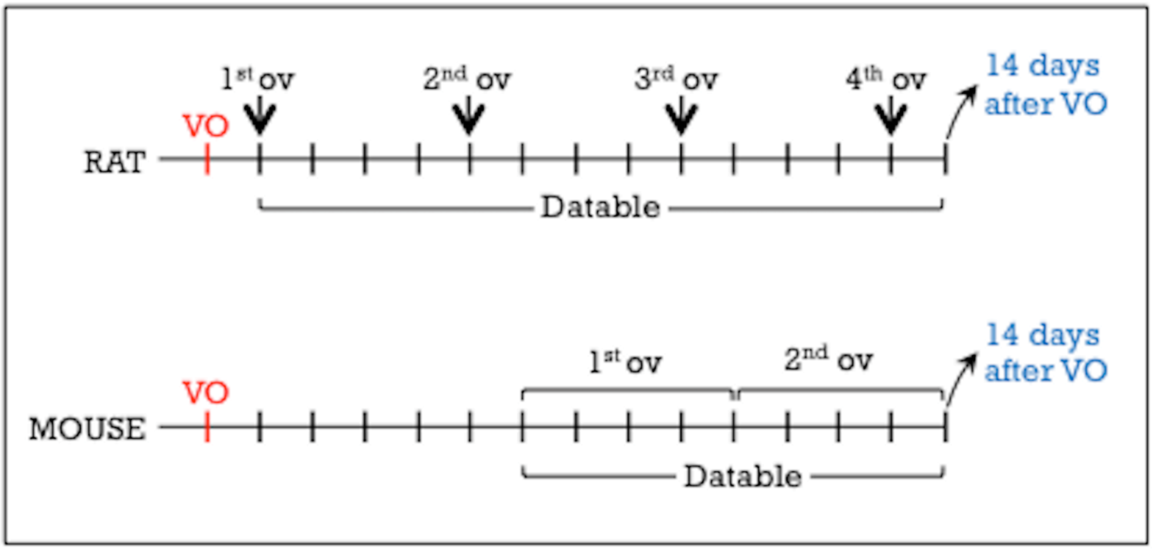

Supplement: Supplementary Figures [file srep46381-s1.pdf]
